# Supplementary material for: Mapping the evolution of fertility support policies in China: A content and instrumental analysis
Source: PLoS One. 2025 Oct 9;20(10):e0332137. doi: 10.1371/journal.pone.0332137 (PMC12510515; doi:10.1371/journal.pone.0332137)
Supplement: S1 Appendix — (ZIP) [file pone.0332137.s001.zip › S1 Appendix. 226 original policy documents/172-中华人民共和国未成年人保护法(2012修正)(FBM-CLI-1-188544).doc]

中华人民共和国未成年人保护法(2012修正)

**发布部门：** [全国人大常委会](https://www.pkulaw.com/law/chl?Aggs.IssueDepartment=201)

**发文字号：**中华人民共和国主席令第65号

**发布日期：**2012.10.26

**实施日期：**2013.01.01

**时效性：** [已被修改](https://www.pkulaw.com/law/chl?Aggs.TimelinessDic=03)

**效力级别：** [法律](https://www.pkulaw.com/law/chl?Aggs.EffectivenessDic=XA0101)

**法规类别：** [老少妇幼残保护](https://www.pkulaw.com/law/chl?Aggs.Category=01604)

**专题分类：** [未成年人保护](https://www.pkulaw.com/law/chl?Aggs.SpecialType=015)

**修改依据：**[中华人民共和国未成年人保护法(2020修订)](javascript:TMP(346976,0,'chl'))

[2012-2020对照](https://www.pkulaw.com/history/compare/对照-3500.html) [2006-2012编注](https://www.pkulaw.com/history/compare/编注-169.html) [1991-2006对照](https://www.pkulaw.com/history/compare/对照-168.html)

中华人民共和国未成年人保护法
（1991年9月4日第七届全国人民代表大会常务委员会第二十一次会议通过　2006年12月29日第十届全国人民代表大会常务委员会第二十五次会议修订 根据2012年10月26日第十一届全国人民代表大会常务委员会第二十九次会议《[关于修改＜中华人民共和国未成年人保护法＞的决定](https://www.pkulaw.com/chl/ff72b2860327707cbdfb.html#class=alink)》修正 主席令第65号）

目录

　　第一章　总则
　　第二章　家庭保护
　　第三章 学校保护
　　第四章　社会保护
　　第五章　司法保护
　　第六章 法律责任
　　第七章 附则

第一章　总则

　　**第一条**　为了保护未成年人的身心健康，保障未成年人的合法权益，促进未成年人在品德、智力、体质等方面全面发展，培养有理想、有道德、有文化、有纪律的社会主义建设者和接班人，根据[宪法](https://www.pkulaw.com/chl/b62e1b65233709b3bdfb.html#class=alink)，制定本法。

　　**第二条**　本法所称未成年人是指未满十八周岁的公民。

　　**第三条**　未成年人享有生存权、发展权、受保护权、参与权等权利，国家根据未成年人身心发展特点给予特殊、优先保护，保障未成年人的合法权益不受侵犯。

　　未成年人享有受教育权，国家、社会、学校和家庭尊重和保障未成年人的受教育权。

　　未成年人不分性别、民族、种族、家庭财产状况、宗教信仰等，依法平等地享有权利。

　　**第四条**　国家、社会、学校和家庭对未成年人进行理想教育、道德教育、文化教育、纪律和法制教育，进行爱国主义、集体主义和社会主义的教育，提倡爱祖国、爱人民、爱劳动、爱科学、爱社会主义的公德，反对资本主义的、封建主义的和其他的腐朽思想的侵蚀。

　　**第五条**　保护未成年人的工作，应当遵循下列原则：

　　（一）尊重未成年人的人格尊严；

　　（二）适应未成年人身心发展的规律和特点；

　　（三）教育与保护相结合。

　　**第六条**　保护未成年人，是国家机关、武装力量、政党、社会团体、企业事业组织、城乡基层群众性自治组织、未成年人的监护人和其他成年公民的共同责任。

　　对侵犯未成年人合法权益的行为，任何组织和个人都有权予以劝阻、制止或者向有关部门提出检举或者控告。

　　国家、社会、学校和家庭应当教育和帮助未成年人维护自己的合法权益，增强自我保护的意识和能力，增强社会责任感。

　　**第七条**　中央和地方各级国家机关应当在各自的职责范围内做好未成年人保护工作。

　　国务院和地方各级人民政府领导有关部门做好未成年人保护工作；将未成年人保护工作纳入国民经济和社会发展规划以及年度计划，相关经费纳入本级政府预算。

　　国务院和省、自治区、直辖市人民政府采取组织措施，协调有关部门做好未成年人保护工作。具体机构由国务院和省、自治区、直辖市人民政府规定。

　　**第八条**　共产主义青年团、妇女联合会、工会、青年联合会、学生联合会、少年先锋队以及其他有关社会团体，协助各级人民政府做好未成年人保护工作，维护未成年人的合法权益。

　　**第九条**　各级人民政府和有关部门对保护未成年人有显著成绩的组织和个人，给予表彰和奖励。

第二章　家庭保护

　　**第十条**　父母或者其他监护人应当创造良好、和睦的家庭环境，依法履行对未成年人的监护职责和抚养义务。

　　禁止对未成年人实施家庭暴力，禁止虐待、遗弃未成年人，禁止溺婴和其他残害婴儿的行为，不得歧视女性未成年人或者有残疾的未成年人。

　　**第十一条**　父母或者其他监护人应当关注未成年人的生理、心理状况和行为习惯，以健康的思想、良好的品行和适当的方法教育和影响未成年人，引导未成年人进行有益身心健康的活动，预防和制止未成年人吸烟、酗酒、流浪、沉迷网络以及赌博、吸毒、卖淫等行为。

　　**第十二条**　父母或者其他监护人应当学习家庭教育知识，正确履行监护职责，抚养教育未成年人。

　　有关国家机关和社会组织应当为未成年人的父母或者其他监护人提供家庭教育指导。

　　**第十三条**　父母或者其他监护人应当尊重未成年人受教育的权利，必须使适龄未成年人依法入学接受并完成义务教育，不得使接受义务教育的未成年人辍学。

　　**第十四条**　父母或者其他监护人应当根据未成年人的年龄和智力发展状况，在作出与未成年人权益有关的决定时告知其本人，并听取他们的意见。

　　**第十五条**　父母或者其他监护人不得允许或者迫使未成年人结婚，不得为未成年人订立婚约。

　　**第十六条**　父母因外出务工或者其他原因不能履行对未成年人监护职责的，应当委托有监护能力的其他成年人代为监护。

第三章　学校保护

　　**第十七条**　学校应当全面贯彻国家的教育方针，实施素质教育，提高教育质量，注重培养未成年学生独立思考能力、创新能力和实践能力，促进未成年学生全面发展。

　　**第十八条**　学校应当尊重未成年学生受教育的权利，关心、爱护学生，对品行有缺点、学习有困难的学生，应当耐心教育、帮助，不得歧视，不得违反法律和国家规定开除未成年学生。

　　**第十九条**　学校应当根据未成年学生身心发展的特点，对他们进行社会生活指导、心理健康辅导和青春期教育。

　　**第二十条**　学校应当与未成年学生的父母或者其他监护人互相配合，保证未成年学生的睡眠、娱乐和体育锻炼时间，不得加重其学习负担。

　　**第二十一条**　学校、幼儿园、托儿所的教职员工应当尊重未成年人的人格尊严，不得对未成年人实施体罚、变相体罚或者其他侮辱人格尊严的行为。

　　**第二十二条**　学校、幼儿园、托儿所应当建立安全制度，加强对未成年人的安全教育，采取措施保障未成年人的人身安全。

　　学校、幼儿园、托儿所不得在危及未成年人人身安全、健康的校舍和其他设施、场所中进行教育教学活动。

　　学校、幼儿园安排未成年人参加集会、文化娱乐、社会实践等集体活动，应当有利于未成年人的健康成长，防止发生人身安全事故。

　　**第二十三条**　教育行政等部门和学校、幼儿园、托儿所应当根据需要，制定应对各种灾害、传染性疾病、食物中毒、意外伤害等突发事件的预案，配备相应设施并进行必要的演练，增强未成年人的自我保护意识和能力。

　　**第二十四条**　学校对未成年学生在校内或者本校组织的校外活动中发生人身伤害事故的，应当及时救护，妥善处理，并及时向有关主管部门报告。

　　**第二十五条**　对于在学校接受教育的有严重不良行为的未成年学生，学校和父母或者其他监护人应当互相配合加以管教；无力管教或者管教无效的，可以按照有关规定将其送专门学校继续接受教育。

　　依法设置专门学校的地方人民政府应当保障专门学校的办学条件，教育行政部门应当加强对专门学校的管理和指导，有关部门应当给予协助和配合。

　　专门学校应当对在校就读的未成年学生进行思想教育、文化教育、纪律和法制教育、劳动技术教育和职业教育。

　　专门学校的教职员工应当关心、爱护、尊重学生，不得歧视、厌弃。

　　**第二十六条**　幼儿园应当做好保育、教育工作，促进幼儿在体质、智力、品德等方面和谐发展。

第四章　社会保护

　　**第二十七条**　全社会应当树立尊重、保护、教育未成年人的良好风尚，关心、爱护未成年人。

　　国家鼓励社会团体、企业事业组织以及其他组织和个人，开展多种形式的有利于未成年人健康成长的社会活动。

　　**第二十八条**　各级人民政府应当保障未成年人受教育的权利，并采取措施保障家庭经济困难的、残疾的和流动人口中的未成年人等接受义务教育。

　　**第二十九条**　各级人民政府应当建立和改善适合未成年人文化生活需要的活动场所和设施，鼓励社会力量兴办适合未成年人的活动场所，并加强管理。

　　**第三十条**　爱国主义教育基地、图书馆、青少年宫、儿童活动中心应当对未成年人免费开放；博物馆、纪念馆、科技馆、展览馆、美术馆、文化馆以及影剧院、体育场馆、动物园、公园等场所，应当按照有关规定对未成年人免费或者优惠开放。

　　**第三十一条**　县级以上人民政府及其教育行政部门应当采取措施，鼓励和支持中小学校在节假日期间将文化体育设施对未成年人免费或者优惠开放。

　　社区中的公益性互联网上网服务设施，应当对未成年人免费或者优惠开放，为未成年人提供安全、健康的上网服务。

　　**第三十二条**　国家鼓励新闻、出版、信息产业、广播、电影、电视、文艺等单位和作家、艺术家、科学家以及其他公民，创作或者提供有利于未成年人健康成长的作品。出版、制作和传播专门以未成年人为对象的内容健康的图书、报刊、音像制品、电子出版物以及网络信息等，国家给予扶持。

　　国家鼓励科研机构和科技团体对未成年人开展科学知识普及活动。

　　**第三十三条**　国家采取措施，预防未成年人沉迷网络。

　　国家鼓励研究开发有利于未成年人健康成长的网络产品，推广用于阻止未成年人沉迷网络的新技术。

　　**第三十四条**　禁止任何组织、个人制作或者向未成年人出售、出租或者以其他方式传播淫秽、暴力、凶杀、恐怖、赌博等毒害未成年人的图书、报刊、音像制品、电子出版物以及网络信息等。

　　**第三十五条**　生产、销售用于未成年人的食品、药品、玩具、用具和游乐设施等，应当符合国家标准或者行业标准，不得有害于未成年人的安全和健康；需要标明注意事项的，应当在显著位置标明。

　　**第三十六条**　中小学校园周边不得设置营业性歌舞娱乐场所、互联网上网服务营业场所等不适宜未成年人活动的场所。

　　营业性歌舞娱乐场所、互联网上网服务营业场所等不适宜未成年人活动的场所，不得允许未成年人进入，经营者应当在显著位置设置未成年人禁入标志；对难以判明是否已成年的，应当要求其出示身份证件。

　　**第三十七条**　禁止向未成年人出售烟酒，经营者应当在显著位置设置不向未成年人出售烟酒的标志；对难以判明是否已成年的，应当要求其出示身份证件。

　　任何人不得在中小学校、幼儿园、托儿所的教室、寝室、活动室和其他未成年人集中活动的场所吸烟、饮酒。

　　**第三十八条**　任何组织或者个人不得招用未满十六周岁的未成年人，国家另有规定的除外。

　　任何组织或者个人按照国家有关规定招用已满十六周岁未满十八周岁的未成年人的，应当执行国家在工种、劳动时间、劳动强度和保护措施等方面的规定，不得安排其从事过重、有毒、有害等危害未成年人身心健康的劳动或者危险作业。

　　**第三十九条**　任何组织或者个人不得披露未成年人的个人隐私。

　　对未成年人的信件、日记、电子邮件，任何组织或者个人不得隐匿、毁弃；除因追查犯罪的需要，由公安机关或者人民检察院依法进行检查，或者对无行为能力的未成年人的信件、日记、电子邮件由其父母或者其他监护人代为开拆、查阅外，任何组织或者个人不得开拆、查阅。

　　**第四十条**　学校、幼儿园、托儿所和公共场所发生突发事件时，应当优先救护未成年人。

　　**第四十一条**　禁止拐卖、绑架、虐待未成年人，禁止对未成年人实施性侵害。

　　禁止胁迫、诱骗、利用未成年人乞讨或者组织未成年人进行有害其身心健康的表演等活动。

　　**第四十二条**　公安机关应当采取有力措施，依法维护校园周边的治安和交通秩序，预防和制止侵害未成年人合法权益的违法犯罪行为。

　　任何组织或者个人不得扰乱教学秩序，不得侵占、破坏学校、幼儿园、托儿所的场地、房屋和设施。

　　**第四十三条**　县级以上人民政府及其民政部门应当根据需要设立救助场所，对流浪乞讨等生活无着未成年人实施救助，承担临时监护责任；公安部门或者其他有关部门应当护送流浪乞讨或者离家出走的未成年人到救助场所，由救助场所予以救助和妥善照顾，并及时通知其父母或者其他监护人领回。

　　对孤儿、无法查明其父母或者其他监护人的以及其他生活无着的未成年人，由民政部门设立的儿童福利机构收留抚养。

　　未成年人救助机构、儿童福利机构及其工作人员应当依法履行职责，不得虐待、歧视未成年人；不得在办理收留抚养工作中牟取利益。

　　**第四十四条**　卫生部门和学校应当对未成年人进行卫生保健和营养指导，提供必要的卫生保健条件，做好疾病预防工作。

　　卫生部门应当做好对儿童的预防接种工作，国家免疫规划项目的预防接种实行免费；积极防治儿童常见病、多发病，加强对传染病防治工作的监督管理，加强对幼儿园、托儿所卫生保健的业务指导和监督检查。

　　**第四十五条**　地方各级人民政府应当积极发展托幼事业，办好托儿所、幼儿园，支持社会组织和个人依法兴办哺乳室、托儿所、幼儿园。

　　各级人民政府和有关部门应当采取多种形式，培养和训练幼儿园、托儿所的保教人员，提高其职业道德素质和业务能力。

　　**第四十六条**　国家依法保护未成年人的智力成果和荣誉权不受侵犯。

　　**第四十七条**　未成年人已经完成规定年限的义务教育不再升学的，政府有关部门和社会团体、企业事业组织应当根据实际情况，对他们进行职业教育，为他们创造劳动就业条件。

　　**第四十八条**　居民委员会、村民委员会应当协助有关部门教育和挽救违法犯罪的未成年人，预防和制止侵害未成年人合法权益的违法犯罪行为。

　　**第四十九条**　未成年人的合法权益受到侵害的，被侵害人及其监护人或者其他组织和个人有权向有关部门投诉，有关部门应当依法及时处理。

第五章　司法保护

　　**第五十条**　公安机关、人民检察院、人民法院以及司法行政部门，应当依法履行职责，在司法活动中保护未成年人的合法权益。

　　**第五十一条**　未成年人的合法权益受到侵害，依法向人民法院提起诉讼的，人民法院应当依法及时审理，并适应未成年人生理、心理特点和健康成长的需要，保障未成年人的合法权益。

　　在司法活动中对需要法律援助或者司法救助的未成年人，法律援助机构或者人民法院应当给予帮助，依法为其提供法律援助或者司法救助。

　　**第五十二条**　人民法院审理继承案件，应当依法保护未成年人的继承权和受遗赠权。

　　人民法院审理离婚案件，涉及未成年子女抚养问题的，应当听取有表达意愿能力的未成年子女的意见，根据保障子女权益的原则和双方具体情况依法处理。

　　**第五十三条**　父母或者其他监护人不履行监护职责或者侵害被监护的未成年人的合法权益，经教育不改的，人民法院可以根据有关人员或者有关单位的申请，撤销其监护人的资格，依法另行指定监护人。被撤销监护资格的父母应当依法继续负担抚养费用。

　　**第五十四条**　对违法犯罪的未成年人，实行教育、感化、挽救的方针，坚持教育为主、惩罚为辅的原则。

　　对违法犯罪的未成年人，应当依法从轻、减轻或者免除处罚。

　　**第五十五条**　公安机关、人民检察院、人民法院办理未成年人犯罪案件和涉及未成年人权益保护案件，应当照顾未成年人身心发展特点，尊重他们的人格尊严，保障他们的合法权益，并根据需要设立专门机构或者指定专人办理。

　　**第五十六条**　讯问、审判未成年犯罪嫌疑人、被告人，询问未成年证人、被害人，应当依照[刑事诉讼法](https://www.pkulaw.com/chl/6d5f42efc8724124bdfb.html#class=alink)的规定通知其法定代理人或者其他人员到场。

　　公安机关、人民检察院、人民法院办理未成年人遭受性侵害的刑事案件，应当保护被害人的名誉。

　　**第五十七条**　对羁押、服刑的未成年人，应当与成年人分别关押。

　　羁押、服刑的未成年人没有完成义务教育的，应当对其进行义务教育。

　　解除羁押、服刑期满的未成年人的复学、升学、就业不受歧视。

　　**第五十八条**　对未成年人犯罪案件，新闻报道、影视节目、公开出版物、网络等不得披露该未成年人的姓名、住所、照片、图像以及可能推断出该未成年人的资料。

　　**第五十九条**　对未成年人严重不良行为的矫治与犯罪行为的预防，依照[预防未成年人犯罪法](https://www.pkulaw.com/chl/1137d4a7728b3300bdfb.html#class=alink)的规定执行。

第六章　法律责任

　　**第六十条**　违反本法规定，侵害未成年人的合法权益，其他法律、法规已规定行政处罚的，从其规定；造成人身财产损失或者其他损害的，依法承担民事责任；构成犯罪的，依法追究刑事责任。

　　**第六十一条**　国家机关及其工作人员不依法履行保护未成年人合法权益的责任，或者侵害未成年人合法权益，或者对提出申诉、控告、检举的人进行打击报复的，由其所在单位或者上级机关责令改正，对直接负责的主管人员和其他直接责任人员依法给予行政处分。

　　**第六十二条**　父母或者其他监护人不依法履行监护职责，或者侵害未成年人合法权益的，由其所在单位或者居民委员会、村民委员会予以劝诫、制止；构成违反治安管理行为的，由公安机关依法给予行政处罚。

　　**第六十三条**　学校、幼儿园、托儿所侵害未成年人合法权益的，由教育行政部门或者其他有关部门责令改正；情节严重的，对直接负责的主管人员和其他直接责任人员依法给予处分。

　　学校、幼儿园、托儿所教职员工对未成年人实施体罚、变相体罚或者其他侮辱人格行为的，由其所在单位或者上级机关责令改正；情节严重的，依法给予处分。

　　**第六十四条**　制作或者向未成年人出售、出租或者以其他方式传播淫秽、暴力、凶杀、恐怖、赌博等图书、报刊、音像制品、电子出版物以及网络信息等的，由主管部门责令改正，依法给予行政处罚。

　　**第六十五条**　生产、销售用于未成年人的食品、药品、玩具、用具和游乐设施不符合国家标准或者行业标准，或者没有在显著位置标明注意事项的，由主管部门责令改正，依法给予行政处罚。

　　**第六十六条**　在中小学校园周边设置营业性歌舞娱乐场所、互联网上网服务营业场所等不适宜未成年人活动的场所的，由主管部门予以关闭，依法给予行政处罚。

　　营业性歌舞娱乐场所、互联网上网服务营业场所等不适宜未成年人活动的场所允许未成年人进入，或者没有在显著位置设置未成年人禁入标志的，由主管部门责令改正，依法给予行政处罚。

　　**第六十七条**　向未成年人出售烟酒，或者没有在显著位置设置不向未成年人出售烟酒标志的，由主管部门责令改正，依法给予行政处罚。

　　**第六十八条**　非法招用未满十六周岁的未成年人，或者招用已满十六周岁的未成年人从事过重、有毒、有害等危害未成年人身心健康的劳动或者危险作业的，由劳动保障部门责令改正，处以罚款；情节严重的，由工商行政管理部门吊销营业执照。

　　**第六十九条**　侵犯未成年人隐私，构成违反治安管理行为的，由公安机关依法给予行政处罚。

　　**第七十条**　未成年人救助机构、儿童福利机构及其工作人员不依法履行对未成年人的救助保护职责，或者虐待、歧视未成年人，或者在办理收留抚养工作中牟取利益的，由主管部门责令改正，依法给予行政处分。

　　**第七十一条**　胁迫、诱骗、利用未成年人乞讨或者组织未成年人进行有害其身心健康的表演等活动的，由公安机关依法给予行政处罚。

第七章　附则

　　**第七十二条**　本法自2007年6月1日起施行。

© 北大法宝：（[www.pkulaw.com](https://www.pkulaw.com/)) 专业提供法律信息、法学知识和法律软件领域各类解决方案。北大法宝为您提供丰富的参考资料，正式引用法规条文时请与标准文本核对。
欢迎查看所有[产品和服务](https://www.pkulaw.com/helps/2.html)。法宝快讯：[如何快速找到您需要的检索结果？](https://www.pkulaw.com/helps/70.html)[法宝](https://www.pkulaw.com/helps/69.html)V6有何新特色？

扫描二维码阅读原文

**原文链接：**<https://www.pkulaw.com/chl/5b242b5a062cc53bbdfb.html>
